# *UHRF1*-mediated epigenetic reprogramming regulates glycolysis to promote progression of B-cell acute lymphoblastic leukemia

**DOI:** 10.1038/s41419-025-07532-0

**Published:** 2025-04-29

**Authors:** Yan Huang, Luting Luo, Yangqi Xu, Jiazheng Li, Zhengjun Wu, Chenxing Zhao, Jingjing Wen, Peifang Jiang, Haojie Zhu, Lingyan Wang, Yanxin Chen, Ting Yang, Jianda Hu

**Affiliations:** 1 Fujian Medical University Union Hospital, Fuzhou, Fujian P.R. China; 2 The Second Affiliated Hospital of Fujian Medical University, Quanzhou, Fujian P.R. China; 3 Institute of Precision Medicine, Fujian Medical University, Fuzhou, Fujian P.R. China; 4 Zhangzhou Affiliated Hospital of Fujian Medical University, Zhangzhou, Fujian P.R. China; 5 Department of Immunology, School of Basic Medical Sciences, Fujian Medical University, Fuzhou, Fujian P.R. China; 6 Department of Lymphoma, Fujian Medical University Cancer Hospital, Fujian Cancer Hospital, Fuzhou, Fujian P.R. China; 7 The Second Department of Hematology, National Regional Medical Center, Binhai Campus of the First Affiliated Hospital, Fujian Medical University, Fuzhou, Fujian P.R. China

**Keywords:** Acute lymphocytic leukaemia, Mechanisms of disease

## Abstract

The prognosis for adult B-cell acute lymphoblastic leukemia remains unfavorable, especially in the context of relapsed and refractory disease. Exploring the molecular mechanisms underlying disease progression holds significant promise for improving clinical outcomes. In this investigation, utilizing single-cell transcriptome sequencing technology, we discerned a correlation between Ubiquitin-like containing PHD and RING finger domain 1 (*UHRF1*) and the progression of B-cell acute lymphoblastic leukemia. Our findings reveal a significant upregulation of UHRF1 in cases of relapsed and refractory B-cell acute lymphoblastic leukemia, thereby serving as a prognostic indicator for poor outcomes. Both deletion of *UHRF1* or overexpression of its downstream target secreted frizzled-related protein 5 (*SFRP5*) resulted in the inhibition of leukemia cell proliferation, promoting cellular apoptosis and induction of cell cycle arrest. Our results showed that *UHRF1* employs methylation modifications to repress the expression of *SFRP5*, consequently inducing the WNT5A-P38 MAPK-HK2 signaling axis, resulting in the augmentation of lactate, the critical metabolic product of aerobic glycolysis. Furthermore, we identified UM164 as a targeted inhibitor of *UHRF1* that substantially inhibits P38 protein phosphorylation, downregulates *HK2* expression, and reduces lactate production. UM164 also demonstrated antileukemic activity both in vitro and in vivo. In summary, our investigation revealed the molecular mechanisms of epigenetic and metabolic reprogramming in relapsed and refractory B-cell acute lymphoblastic leukemia and provides potential targeted therapeutic strategies to improve its inadequate prognosis.

**Figure:**
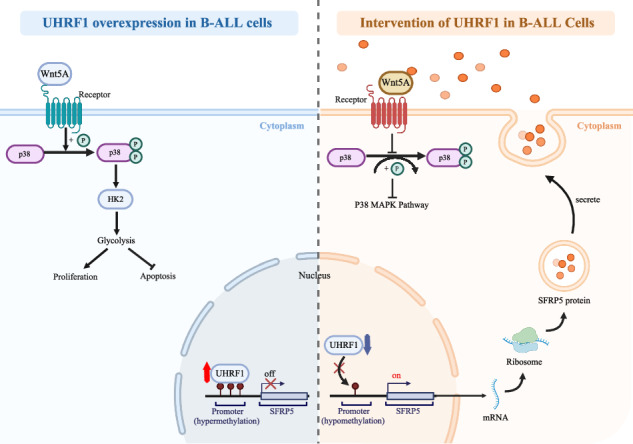
The schematic model showed the regulator network of UHRF1-SFRP5-WNT5A-P38 MAPK-HK2 in B-ALL.

The schematic model showed the regulator network of UHRF1-SFRP5-WNT5A-P38 MAPK-HK2 in B-ALL.

## Introduction

Acute lymphoblastic leukemia (ALL) is a malignant clonal hematopoietic disorder that arises from hematopoietic stem cells or progenitor cells and exhibits considerable heterogeneity. B-cell acute lymphoblastic leukemia (B-ALL) accounts for 80% of pediatric leukemia, and approximately 75% in adult ALL [1]. Despite advancements in chemotherapy and cellular immunotherapy, the prognosis of adult ALL remains poor [2]. Although a substantial number of patients achieve complete remission (CR) following standard chemotherapy, ~50% experience relapse [3]. Relapsed ALL often leads to resistance to chemotherapy, resulting in refractory leukemia. The median overall survival (OS) of patients with refractory leukemia is typically only 3–6 months [4].

Currently, effective and safe curative strategies for the treatment of ALL are lacking. Despite the ability of cellular immunotherapy to achieve high remission rates, such as chimeric antigen receptor T (CAR-T) cell therapy and CD19 monoclonal antibodies, issues of disease relapse and adverse side effects persist [5, 6]. High relapse and refractory rates are major causes of treatment failure in patients with B-ALL. Hence, further investigations are needed to explore the molecular mechanisms underlying relapse and refractory events, as well as to identify potential therapeutic targets.

The mechanisms of refractory and relapse of leukemia are extremely complex and include clonal evolution, primary or acquired drug resistance, and immune evasion [7, 8]. Drug resistance is a notable challenge in oncotherapy, and its precise mechanisms are not fully understood. While drug resistance in tumors has traditionally been attributed to irreversible genetic mutations, emerging evidence suggests that epigenetic reprogramming plays a substantial role in the development of resistance [9]. ALL is characterized by a markedly high level of DNA methylation [10]. DNA methylation plays a pivotal role in the pathogenesis, treatment outcomes, and refractory relapse of ALL [11]. Aberrant DNA methylation is closely associated with higher relapse rates and shorter OS, and demethylation reversal can facilitate apoptosis of ALL cells and enhance their sensitivity to chemotherapeutic agents [12]. However, clinical trials provide very limited evidence of the effectiveness of DNA methyltransferase inhibitors in treating patients with ALL [13]. Hence, the mechanism of DNA methylation modification in the progression of ALL needs to be further explored.

This study aimed to analyze the molecular genetic changes in patients with B-ALL from the initial diagnosis to the relapsed and refractory phases. Single-cell transcriptome sequencing (scRNA-seq) was performed to characterize the gene expression profiles across patients with distinct disease states of B-ALL and healthy individuals.

## Methods

### Cell lines

All cell lines were authenticated by short tandem repeat (STR) profiling to confirm their identity. Additionally, the testing for mycoplasma contamination was negative prior to experimentation. The human leukemia cell lines Nalm6 (N6), Nalm6-Luciferase (N6-Luc), Reh, MV4-11, Kasumi, Jurkat, BALL-1 and Molt4, were cultivated in RPMI 1640 medium supplemented with 10% FBS. Hmy2.CIR cells were cultured in IMEM supplemented with 10% FBS. HEK-293T cells were cultured in DMEM containing 10% FBS. All cell lines were incubated at 37 °C in an environment of 5% CO_2_ and 95% humidity, in a dedicated cell incubator. Cells in the logarithmic growth phase were used for subsequent experiments.

### Patients and healthy donors sample isolation

The study involving human participants was conducted according to the Declaration of Helsinki and approved by the Institutional Review Board of the Union Hospital of Fujian Medical University (2021KJCX033). Written informed consent was obtained from all participants prior to their inclusion in the study. A total of 90 B-ALL patient samples, including 31 relapsed cases, 48 newly diagnosed cases, and 11 CR cases, were collected from 84 patients treated at the Union Hospital of Fujian Medical University (Fig. S1A). These comprised 66 bone marrow specimens and 24 peripheral blood samples (Fig. S1B). Among these, 5 patients contributed samples at both the newly diagnosed and relapsed stages, while 1 patient provided samples at both the relapsed and remission stages. Additionally, peripheral blood samples from 25 healthy donors were included as controls in this study. Mononuclear cells were isolated from bone marrow or peripheral blood cells using Lymphoprep reagent (Cat.#18061, STEMCELL Technologies, Vancouver, Canada). CD19^+^ cell sorting was performed using the EasySep Release Human CD19 Positive Selection Kit (Cat.#17854, STEMCELL Technologies, Vancouver, Canada), following the guidelines provided by the manufacturer.

### Survival analysis

This study included 51/74 adult B-cell ALL patients who had received at least two rounds of chemotherapy, had not been lost to follow-up, and had available samples from either their initial diagnosis or relapse (Fig. S1A). Clinical data for these patients was collected from our center. Additionally, the mRNA expression dataset and clinical information for 369 pediatric B-cell ALL patients were obtained from the TARGET database. The optimal cut-off value for *UHRF1* expression was determined using the log-rank test based on R package “survminer,” and Kaplan–Meier analysis was employed to assess OS.

### Plasmid construction and transfection in B-ALL cell lines

The LentiCRISPR v2 plasmid (Cat #52961) was purchased from Addgene (Cambridge, USA). Specific guide RNAs, designed to target UHRF1, as well as non-targeting guide RNAs were generated using the GPP Web Portal online tool [14, 15]. Plasmids were transfected into HEK-293 T cells to package the lentivirus, using Lipofectamine 3000 reagent (Cat.# L3000015, Thermo Fisher Scientific, Waltham, USA) according to the manufacturer’s protocol. Non-targeting gRNA were used as controls. After 48 h, the target cells were sorted using puromycin (Cat.# 58-58-2, Merck & Co., Inc., Kenilworth, USA) until the knockout of UHRF1. To construct plasmids for UHRF1 restoration, the knockout sequence was subjected to synonymous mutations, synthesized, and cloned into the pCDH-CMV-MCS-EF1a-mCherry vector (BioSune Biotechnology, Shanghai, China). An empty vector was used as the control. Viral packaging and cell transfection were performed as described previously [16]. The oligos were shown as follows:

UHRF1-sgRNA1: 5ʹ-GCGGGAACTCTACGCCAACG-3ʹ;

UHRF1-sgRNA2: 5ʹ-CCATACCCTCTTCGACTACG-3ʹ;

Nontarget-sgRNA: 5ʹ-CGCACGACCATTGCTGCTGC-3ʹ;

UHRF1-KO-Ve: 5ʹ-GCGGGAACTCTACGCCAACG- 3ʹ;

UHRF1-KO-Re: 5ʹ-TCGAGAGCTGTATGCTAATG- 3ʹ.

### Single-cell RNA sequencing (scRNA-seq)

scRNA-seq was performed using a 10X Chromium system. Single-cell capture, cDNA synthesis, and amplification were performed according to the manufacturer's instructions. Libraries were constructed using the single-cell 3’ gene expression profiling library preparation and the Gel Bead V3 reagent kit (10X Genomics, San Francisco, USA). Subsequently, the libraries were sequenced on an Illumina NovaSeq6000 sequencer, with a sequencing depth of at least 100,000 reads per cell, employing a paired-end 150 bp read strategy. Cells with a gene count of less than 200 or cells with a mitochondrial gene ratio >25% were considered low-quality cells and were subsequently filtered out using the Seurat 3.0R package. Principal component Analysis (PCA) was performed for dimensionality reduction, followed by visualization using the uniform manifold approximation and projection (UMAP) method. The cell types were annotated using the CellMarker and singleR R packages.

### RNA extraction and real-time quantitative reverse transcription PCR (RT-qPCR)

Total RNA was extracted using the TRIzol reagent (Cat.#15596026, Thermo Fisher Scientific, Waltham, USA), followed by cDNA preparation using a reverse transcription reagent kit (Cat.#A5001, Promega Corporation, Madison, USA). RT-qPCR was performed using the SYBR Green Supermix (Cat.#Q711-03, Vazyme Biotechnology, Shanghai, China) on an Applied Biosystems 7500 Real-Time PCR System. The primer sequences used were as follows:

18S-F: 5′-GACACGGACAGGATTGACAGATTG-3′;

18S-R: 5′-TGCCAGAGTCTCGTTCGTTATCG-3′;

UHRF1-F: 5′-GGAGCGTACTCCCTAGTCCT-3′;

UHRF1-R: 5′-CCCTGTTGGTGTTGGTGAGT-3′;

SFRP5-F: 5′-GTGCTCCAGTGACTTTGTGG-3′;

SFRP5-R: 5′-CCGCGCCATTCTTCATGTG-3′;

ENO1-F: 5′-AAGGCCGTGAACGAGAAGTC-3’;

ENO1-R: 5′-CCCGAACGATGAGACACCAT-3’;

HK2-F: 5′-TTGACCAGGAGATTGACATGGG-3’;

HK2-R: 5′-CAACCGCATCAGGACCTCA-3’.

### Western blotting

Total protein extracts for immunoblotting were prepared by incubating the cells for 30 min in RIPA lysis buffer (Cat.#PC101, Shanghai Epizyme Biomedical Technology Co., China) with ultrasonic disruption. The Bio-Rad western blotting workflow was used for immunoblotting. After blocking in 5% non-fat milk, the nitrocellulose membranes were incubated with primary and secondary antibodies. The antibodies used were: *UHRF1* (Cat.#A2343, ABclonal Biotechnology, Wuhan, China), *SFRP5* (Cat.#A16734, ABclonal Biotechnology, Wuhan, China), *HK2* (Cat.#A22319, ABclonal Biotechnology, Wuhan, China), P38 (Cat.#8690S, Cell Signaling Technology, Danvers, USA), P-P38 (Cat.#4511S, Cell Signaling Technology, Danvers, USA), α-Tubulin (Cat.# PTM-7176, Jingjie PTM BioLab Co., Inc., Hangzhou, China) and β-actin (Cat.#AC026, ABclonal Biotechnology, Wuhan, China). Signals were detected with an enhanced chemiluminescence reagent (Cat.#32106, Thermo Fisher Scientific, Waltham, USA) using ChemiDoc Imagers (Bio-Rad, USA).

### Apoptosis, cell proliferation, and cell cycle assays

Samples were stained with Annexin-V (Cat.#640920, BioLegend, San Diego, USA) and 7-AAD (Cat.#420404, BioLegend, San Diego, USA) to test for cell apoptosis using flow cytometry. Cell viability was determined using MTT (Cat.#IM0280, Solarbio, Beijing, China) or MTS reagent (Cat.#G3580, Promega Corporation, Madison, USA). Absorbance was measured using a spectrophotometer (STAT FAX-2100) at a wavelength of 490 nm. Cells were fixed in 75% ethanol at −20 °C overnight before the cell cycle assay and then were stained with PI (BD, USA) for flow cytometry.

### RNA sequencing

Total RNA was subjected to next-generation sequencing (NGS) using the Illumina MiSeq. The integrity and quality of RNA were determined on the Bioanalyzer 2100 (Agilent Technologies, Santa Clara, USA) and RNA 6000 Nano Lab Chip (Agilent Technologies, Santa Clara, USA). Then, 3 µg of total RNA was used for library preparation with the NEBNext Ultra II RNA Library Prep Kit (Cat.#E7775S, New England Biolabs, Ipswich, USA) designed for Illumina sequencing. The raw data in fastq format were processed using Fastp software (version 0.19.3, HaploX Biotechnology, China). Clean reads were obtained by removing the reads containing adapters, poly N sequences, and low-quality bases. All subsequent downstream analyses were performed exclusively using clean high-quality data. The reference genome and gene model annotation files were downloaded directly from the Genome website (https://www.ncbi.nlm.nih.gov/assembly/GCF_000001405.26/). Paired-end clean reads were aligned to the reference genome using STAR V20201, a widely used RNA-Seq read aligner. Cufflinks v2.2.1 was utilized to compute the read counts corresponding to each gene. Subsequently, the fragments per kilobase of transcript per million mapped reads (FPKM) for each gene were calculated based on its length, and genes were subjected to read-length counting. DEG analysis between groups was performed using the DESeq2 package in R. The resulting *p* values were adjusted using the Benjamini and Hochberg method to control the FDR. Gene Ontology (GO) and KEGG enrichment analyses of DEGs were performed using Cluster Profiler. All statistical analyses and plotting in this section were performed using R (version 4.0.3).

### 850K Methylation BeadChip array

Genomic DNA was isolated using a QIAamp DNA Blood Mini Kit (Cat.# 51104, Qiagen, Hilden, Germany) for DNA methylation analysis. Bisulfite conversion was performed using the EZ DNA Methylation-Gold kit (Cat.# D5005, Zymo Research, Irvine, USA) according to the manufacturer’s protocol. The converted DNA was hybridized to an Illumina Infinium 850 K array chip, followed by overnight incubation in a dedicated hybridization oven. DNA captured on the chip served as a template for single-base extension reactions. Fluorescently detectable tags were used to determine the methylation status of the samples. The chip was scanned to generate raw data, which was subsequently analyzed using the GenomeStudio 2.0 data analysis software (Illumina, San Diego, USA). After quality filtering and data normalization, *β*-value (Δ*β*) analysis was performed using the BMIQ method to evaluate the methylation levels for each CpG site. CpG sites that showed |delta *β*| > 0.1 and adjusted *p* values < 0.05 were defined as significant methylation variable positions (MVPs). Additionally, the Probe Lasso method in the ChAMP R package was used to detect DMRs.

### BSP

Total DNA extraction followed by bisulfite modification was performed according to established protocols. For the subsequent analysis of the methylation status of the SFRP5 CpG Island, bisulfite sequencing PCR primers designed by BioSune (China) were used. The primer sequences utilized were as follows:

SFRP5-BSP-F: 5′-ATGGGGTTTGGTATTAAGTTTAATG-3′;

SFRP5-BSP-R: 5′-ACTCCCTACCTCCCTAAACATTTT-3′.

Upon successful amplification of the DNA promoter region of SFRP5, the PCR products were subsequently purified and cloned for sequencing using the pUC18-T vector (Cat.#B300692, Sangon Biotech, Shanghai, China). Finally, the obtained single clone sequences were thoroughly analyzed and visualized using the BiQ Analyzer software (Version 2.0).

### CHIP-qPCR

Chromatin immunoprecipitation (ChIP) was performed using a Pierce Magnetic ChIP Kit (Cat.#26157, Thermo Fisher Scientific, Waltham, USA) as per the manufacturer’s instructions. Briefly, the samples were finely minced and crosslinked with 1% formaldehyde for 15 min at room temperature, followed by prompt neutralization with glycine for 5 min. The sonication was used to both disrupt the nuclear membrane and fragment the chromatin (using a cycle of 195 W, for 2 s followed by 24 s off for a total of six cycles). For the immunoprecipitation step, the chromatin fragments were incubated overnight at 4 °C with rabbit anti-UHRF1 (Cat.#NBP2-20807, Novus Biologicals) or rabbit IgG (Cat.#26157, Thermo Fisher Scientific, Waltham, USA) antibodies, followed by incubation for 2 h with Protein A/G Magnetic Beads. After thoroughly washing the immune complexes, the eluted DNA was evaluated by RT-qPCR. The results were calculated as a percentage of input DNA (% input). The primer sequences utilized are as follows:

SFRP5-D-F1: 5′-TCACCCCTCTCCACTACCAC-3′;

SFRP5-D-R1: 5′-GCAGGAGTACTGGGCACTTT-3′;

SFRP5-D-F2: 5′-CCCACTGTGTTCCCTACCAT-3′;

SFRP5-D-R2: 5′-AGGAGGCCTGGATTCTGAGT-3′.

### Metabolomics analysis

The non-targeted medium and cell metabolite analyses were performed by NMR spectroscopy. The ^1^H NMR spectra were acquired at 298 K using a Bruker Advance III 600 spectrometer (Bruker BioSpin, Germany) operating at a ^1^H frequency of 600.13 MHz. Spectral data in the chemical shift range of 0.6–9.00 ppm was selected, integrated, and normalized. Finally, the NMR spectra were transformed into a data list by following the below steps.

Targeted metabolites were detected using MetWare (http://www.metware.cn/), based on AB Sciex, with six biological replicates. Significantly regulated metabolites between the groups were determined by Variable Importance in Projection (VIP) and absolute Log2 fold change. VIP values were extracted from the OPLS-DA results, which also contained score plots and permutation plots, and were generated using the R package MetaboAnalystR. The data were log-transformed (log2) and mean-centered before OPLS-DA was performed. A permutation test (200 permutations) was performed to avoid overfitting.

Lactate level was detected by colorimetric kit (Cat.#E-BC-K044-M, Elabscience, Wuhan, China), following the provided protocol.

### Measurement of extracellular acidification rate (ECAR)

The ECAR of cells was assessed using the Seahorse XFe24 Flux Analyzer (Agilent, Santa Clara, USA) and SpectraMax iD5 Multi-Mode Microplate Reader (Molecular Devices, Sunnyvale, USA). The glycolytic rate assay kit (Cat.#103020–100, Agilent, Santa Clara, USA) and extracellular acidification kit (Cat.#BB-48311, Bestbio, Shanghai, China) were used to measure ECAR. For the glycolytic rate assay, cells were seeded in a 24-well XF Seahorse incubation plate at a density of 1 × 10^5^ cells per well, following the provided protocol. The cells were cultured at 37 °C in XF base medium (pH 7.4), and glucose (10 mM), oligomycin (1 μM), and 2-DG (50 mM) were sequentially added to the plates at different time points according to the manufacturer’s instructions. To measure ECAR using the extracellular acidification kit, 1 × 10^6^ cells were seeded into 96-well black-bottom flat plates and incubated for at least three hours in a 37 °C incubator without CO_2_. The cells were then incubated with the BBcellProbe® P61 probe, and the plates were analyzed using a microplate reader at 37 °C for 120 min, with measurements taken every three minutes (Ex488/Em580). The ECAR was calculated as the ratio of fluorescence difference between two measurements to the time interval.

### Immunofluorescence microscopy

Cells were fixed for 15 min in 4% paraformaldehyde (Cat.#P1110, Solarbio, Beijing, China), washed 3 times for 5 min each with 1×PBS, permeabilized with 0.1% Triton X-100 for 10 min at room temperature (Cat.#T8200, Solarbio, Beijing, China), blocked with 1% bovine serum albumin (Cat.#PS113, Shanghai Epizyme Biomedical Technology Co., China) for 60 min, and incubated overnight at 4 °C with the SFRP5 and WNT5A primary antibody (1:300, Cat.#A19133, ABclonal Biotechnology, Wuhan, China). The cells were incubated with anti-Rabbit IgG Alexa Fluor®-594 secondary antibody (Cat.#GB28301, Servicebio, China) at room temperature. Then nuclei were stained with DAPI (Servicebio, China) in the dark for 10 min. Finally, cells were observed with a Leica fluorescence microscope (Leica, German).

### Biolayer interferometry (BLI) assay

The biotinylated SFRP5 protein was immobilized on the probe-based biosensor (ForteBio Octet RED 96e, USA). The WNT5A protein was diluted in Assay Buffer to concentrations ranging from 0.156 to 5 μM. After Baseline Stabilization, the biosensor tips were dipped into solutions containing various concentrations of the WNT5A protein to measure the association phase. Then, the biosensor tips were dipped back into the running buffer to measure the dissociation phase. The changes were monitored in signal as WNT5A binds to and dissociates from the immobilized SFRP5. The binding affinity (KD) and concentration information were calculated and analyzed using the Global fitting model.

### Screening of small molecule inhibitors of UHRF1

A virtual screening campaign was conducted using the Schrödinger Maestro Software (version 11.4). The 3D structure of Human UHRF1 (Protein Data Bank [PDB] ID: 6VCS) was obtained from the RCSB PDB database and imported into the system. To ensure structural integrity, the proteins were hydrogenated using the Protein Preparation Wizard module. The MCE Bioactive Compound Library, containing 17,200 compounds in a 2D format, was used as the input. These compounds underwent a comprehensive transformation process using the LigPrep Module in the Schrödinger software. This procedure involves hydrogenation, energy optimization, and other essential transformations to generate 3D structures in preparation for subsequent virtual screening. Virtual screening was performed using a virtual screening workflow module. Within this framework, the prepared compounds were imported and subjected to molecular docking analysis using the Glide module. The docking process engendered geometric and energetic congruence between the receptor and ligand entities, facilitating potential molecular interactions. A high-throughput screening mode within the Glide module was employed in the first round of screening. The compounds exhibiting scores within the upper 15th percentile were earmarked for the secondary screening phase using the standard mode. Subsequently, the top 15% of the compounds underwent a tertiary screening phase employing a high-precision mode, culminating in the final ranking of small-molecule compounds. The top 200 compounds were selected as candidate UHRF1 inhibitors.

### Reagent specifications

All chemical reagents were obtained from commercial sources with the following identifiers: UM164 (Cat#S8706, Selleck Chemicals, USA); SB203580 (Cat#HY-10256, MedChemExpress, USA); BOX5 (Cat#P1216, Selleck Chemicals, USA)). All compounds were reconstituted according to manufacturer protocols, and purity (>98%) was validated prior to experimental use.

### Microscale thermophoresis (MST)

MST experiments were performed to confirm the binding affinity of the ligand (UM164) for the target protein (UHRF1). The pCDNA3.4 expression vector carrying the genetic sequence of the *UHRF1* gene was transfected into HEK293 cells. Following transfection, protein extraction was conducted, and the protein expression status was determined by western blot analysis. After validation, the target protein was purified using Ni-resin affinity chromatography, resulting in the isolation of the desired protein with purity exceeding 80%. UM164 (1 mM) was diluted two-fold to generate a series of 16 graded concentrations. Subsequently, the UHRF1 protein underwent fluorescence labeling using the Monolith RED-NHS kit (Nano Temper, Germany). This labeled protein was then diluted to achieve a final concentration of 1 µM. The final protein concentration was combined with a solution of small molecule compounds to establish a binding reaction system. The mixture was incubated at room temperature for 30 min, followed by loading onto Monolith NT.115 standard-coated capillaries (Nano Temper, Germany) for analysis.

### Cell line-derived xenograft (CDX) transplantation model and treatment

We procured 6-week-old male NOD/SCID/IL2Rγ-null (NSG) mice from the Shanghai Model Organisms Center, Inc. (Shanghai, China) and maintained them under specific-pathogen-free (SPF) conditions. A total of 2 × 10^4^ Nalm6-Luc cells were intravenously injected into mice via the tail vein to establish a human B-ALL xenograft model. The In Vivo Imaging System (IVIS; PerkinElmer, Germany) was used to assess tumor burden in mice. Based on the imaging results on day 5, we evenly divided the mice into control and treatment groups to ensure comparable baseline disease levels between groups. The antitumor effect of the *UHRF1* inhibitor UM164 in vivo was evaluated as follows: At 5-day post-xenotransplantation, UM164 (10 mg/kg) or the vehicle control was injected intraperitoneally (i.p.) every other day for 14 days. Peripheral blood samples were analyzed ~21 days after transplantation to detect human leukemic cells via flow cytometry. Leukemia progression was also evaluated using H&E and immunohistochemical staining. Clinical conditions and body weight were monitored regularly to identify potential drug toxicity.

### Statistics

GraphPad Prism (version 8.0) was used for statistical analyses. Data were expressed as means ± SD of the mean. The results were obtained from at least three independent experiments. Unpaired or paired two-tailed Student’s *t*-tests were used for comparisons between two groups, whereas ordinary one-way ANOVA was used for comparisons involving more than two groups. The Kaplan–Meier method was used to generate survival curves, which were compared using the log-rank test. The level of significance was set at *p* < 0.05.

## Results

### ScRNA-seq reveals the involvement of *UHRF1* in the progression of B-ALL

**Fig. 1:**
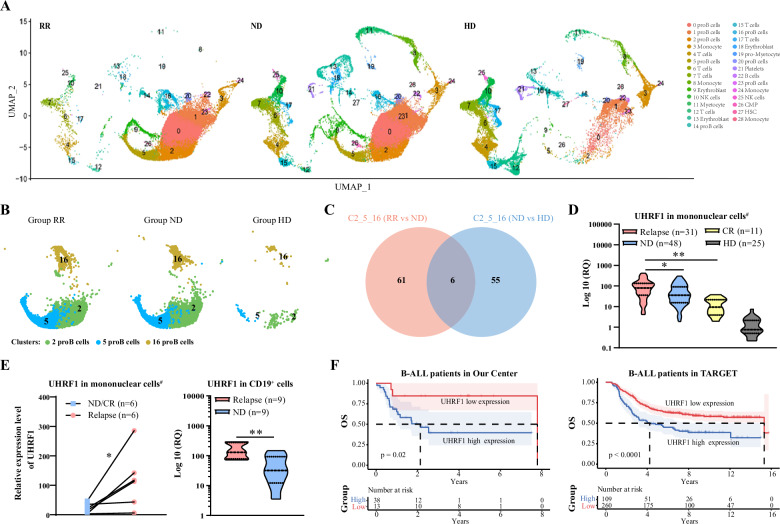
Sc-RNAseq analysis of B-ALL patients. **A** Immunophenotyping of single-cell transcriptomes from patients with different disease states of B-ALL and HD. **B** UMAP diagram illustrates the distribution of clusters 2, 5, and 16 across different groups. **C** The Venn diagram revealed six significantly common DEGs. **D** The mRNA expression levels of UHRF1 in B-ALL Patients across varied disease states. **E** Paired analysis of mRNA expression levels of UHRF1 in mononuclear cells at relapse state vs. ND/CR state (left); Comparative analysis of mRNA expression levels of UHRF1 in CD19^+^ B cells (right). **F** Survival analysis of UHRF1 in B-ALL Patients within our center and the TARGET dataset. proB: B progenitor cells, RR: Relapsed/Refractory group, ND: Newly Diagnosed group, CR: Complete Remission group, HD: Healthy Donor group. ^#^Mononuclear cells: including bone marrow mononuclear cells (BMMNCs) and peripheral blood mononuclear cells (PBMCs).

To elucidate the molecular mechanisms underlying the progression of B-ALL, we collected peripheral blood and bone marrow samples from nine individuals for scRNA-seq using a 10× single-cell platform. Among these samples, six were from patients with B-ALL (three with relapsed and refractory (RR) and three were newly diagnosed (ND)) and three were from healthy donors (HD). Of the RR samples, two were derived from peripheral blood and one from bone marrow. Similarly, among the ND samples, two were obtained from peripheral blood and one from bone marrow. All samples from healthy donors were collected from peripheral blood. By employing dimensionality reduction techniques and clustering methodologies, a comprehensive compilation of 29 discrete cellular subpopulations (clusters) was successfully identified. Referring to the human primary cell atlas (HPCA) dataset, cell type annotation was performed and the results were illustrated in Fig. 1A, predominantly encompassing eight subclusters of progenitor B cells (clusters 0, 1, 2, 5, 14, 16, 20, 23), one cluster of B cells (cluster 22), six clusters of T cells (clusters 4, 6, 7, 12, 15, 17), two clusters of NK cells (clusters 10, 25), three clusters of monocytes (clusters 3, 24, 28), two clusters of neutrophils (clusters 8, 11), and three clusters of primitive erythrocytes (clusters 9, 13, 18). As shown in Fig. 1A, the single-cell landscapes exhibited differences among the three groups. The results showed that the proportions of the three subgroups (clusters 2, 5, and 16) exhibited a decreasing trend in the progenitor B cell population in the RR, ND, and HD groups (Figs. 1B and S1D).

Subsequently, differential expression analysis among the three progenitor B cell subgroups (clusters 2, 5, and 16) was conducted. Significant differentially expressed genes (DEGs) were defined by the criterion of average log2| Foldchange | > 0.5 and *p*-values < 0.05 were defined as significant DEGs, 67 and 61 DEGs were identified in the RR vs. ND group and the ND vs. HD group, respectively. To further select the key DEGs within clusters 2, 5, and 16, six significantly common DEGs were identified (*UHRF1*, *IGFBP2*, *S100A16, S100A13, CCL17* and *RAB13;* Fig. 1C), potentially exhibiting increasing or decreasing trends among the RR, ND, and HD groups.

To confirm the critical involvement of common DEGs in B-ALL, a total of 90 peripheral blood and bone marrow specimens were collected from 84 B-ALL patients treated at our center. This included 12 samples from 6 patients at different disease stages, with 5 patients providing both newly diagnosed and relapsed samples, and 1 patient providing both relapsed and remission samples (Fig. S1B). Specifically, the dataset comprised 31 relapse samples (22 bone marrow and 9 peripheral blood), 48 newly diagnosed samples (36 bone marrow and 12 peripheral blood), and 11 complete remission samples (8 bone marrow and 3 peripheral blood). Peripheral blood samples were collected from 25 healthy donors for comparative analysis.

The results of RT-qPCR showed that *UHRF1* exhibited the highest expression levels in RR patients followed by ND patients, both of which were significantly higher than those in CR patients and HD (Figs. 1D and S1C). However, the remaining 5 genes showed no significant difference between the groups (Fig. S1E). Western blot experiments also demonstrated a significant upregulation of *UHRF1* protein levels in B-ALL patients compared to those in HD patients (Figs. S1F and S4G). Additionally, in paired samples, *UHRF1* expression levels in relapsed patients were higher than those in the ND or CR groups (Fig. 1E). To further elucidate the differential expression of *UHRF1* in CD19-positive B cells from patients with distinct pathological conditions, we used magnetic bead-based cell sorting to isolate CD19-positive cells and investigated the expression of *UHRF1* at the mRNA level. The findings revealed substantially elevated *UHRF1* expression in CD19^+^ B cells from relapsed patients compared with those in ND patients (Fig. 1E).

To investigate the potential link between aberrant *UHRF1* expression and the prognosis of patients with B-ALL, a comprehensive survival analysis was conducted on cohorts of 51 patients with B-ALL from our institution’s dataset. The patients’ clinical characteristics are summarized in Table 1. The results showed that, compared to the low-expression group, the proportion of patients aged 35 years or older was significantly higher in the *UHRF1* high-expression group (*P* = 0.028). Additionally, the proportion of patients who did not achieve CR was higher, though the difference was not statistically significant (*P* = 0.051). No other differences in baseline clinical characteristics were observed. The median follow-up period was 2.5 years. A log-rank test was used to determine the optimal cutoff value for UHRF1 mRNA expression levels measured by the RT-qPCR method, using the average expression level in healthy donors (HD) as a reference. Patients with UHRF1 expression levels exceeding a relative quantification (RQ) value of 21 were classified as the high-expression group (*n* = 38), while those with levels ≤ 21 were categorized as the low-expression group (*n* = 13, Fig. S1G). Survival analysis indicated that OS was significantly lower in the *UHRF1* high-expression group than that in the low-expression group in our center (5-year OS was 39.7% vs. 84.6%, 95% CI was 24.3–64.8% vs. 67.1–100%, *P* = 0.02, Fig. 1F). The results were verified using the publicly accessible TARGET database (https://ocg.cancer.gov/programs/target), which includes 369 patients with B-ALL. The log-rank statistic was used to determine the optimal cutoff value based on microarray data (Fig. S1H). Patients with relative mRNA expression levels of *UHRF1* ≥ 13.24, normalized and log2-transformation, were categorized as the high-expression group (*n* = 109), while those with levels below this threshold were classified as the low-expression group (*n* = 260). Survival analysis also indicated that the group with high *UHRF1* expression had a lower OS compared to the group with low expression (5-year OS was 48.5% vs. 65.5%; 95% CI, 39.9-59.1% vs. 59.9-71.6%, *P* < 0.0001, Fig. 1F). These findings suggest the involvement of *UHRF1* in the pathogenesis of B-ALL, thereby presenting *UHRF1* as a novel therapeutic target.

**Table 1:** The clinical characteristics of patients in our central.

Characteristics	Number	*UHRF1* expression		*P*
		Low (%)	High (%)	
Cases	51	13	38	
*Age*				
<35 years	22	9 (69.2)	13 (34.2)	0.028
≥35 years	29	4 (30.8)	25 (65.8)	
*Gender*				
Female	28	6 (46.2)	22 (57.9)	0.463
Male	23	7 (53.8)	16 (42.1)	
*WBC* (×10^9^/*L*)				
<30	30	10 (76.9)	20 (52.6)	0.125
≥30	21	3 (23.1)	18 (47.4)	
*BCR-ABL*				
Negative	31	10 (76.9)	21 (55.3)	0.167
Positive	20	3 (23.1)	17 (44.7)	
*Complete remission (CR)*				
No	6	0 (0.0)	6 (15.8)	0.051
Yes	45	13 (100.0)	32 (84.2)	
*Time to achieve CR*				
≤4 week	41	10 (76.9)	31 (81.6)	0.719
>4 weeks	10	3 (23.1)	7 (18.4)	
*Cytogenetic risk stratification*				
Non-favorable	16	7 (53.8)	28 (73.7)	0.192
Favorable	35	6 (46.2)	10 (26.3)	
*Transplantation*				
Yes	22	8 (61.5)	14 (36.8)	0.121
No	29	5 (38.5)	24 (63.2)	

### *UHRF1* affects cell proliferation and apoptosis in B-ALL cells

The Cancer Cell Line Encyclopedia (CCLE) database was explored to elucidate the expression patterns of *UHRF1* in different tumor cell lines. High mRNA expression levels of *UHRF1* were most pronounced in B-lymphoblastic leukemia/lymphoma cell lines (Fig. S2A). Western blot results showed higher protein expression levels of UHRF1 in Reh and Nalm6 cells compared to other leukemia cell lines (BALL-1, Jurkat, Molt4, MV4-11, and Kasumi), as well as in the normal B lymphoblastoid cell line HMy2.CIR (Figs. S2B and S4H). Therefore, Reh and Nalm6 cell lines were selected as cellular models to study the functional significance of *UHRF1*.

**Fig. 2:**
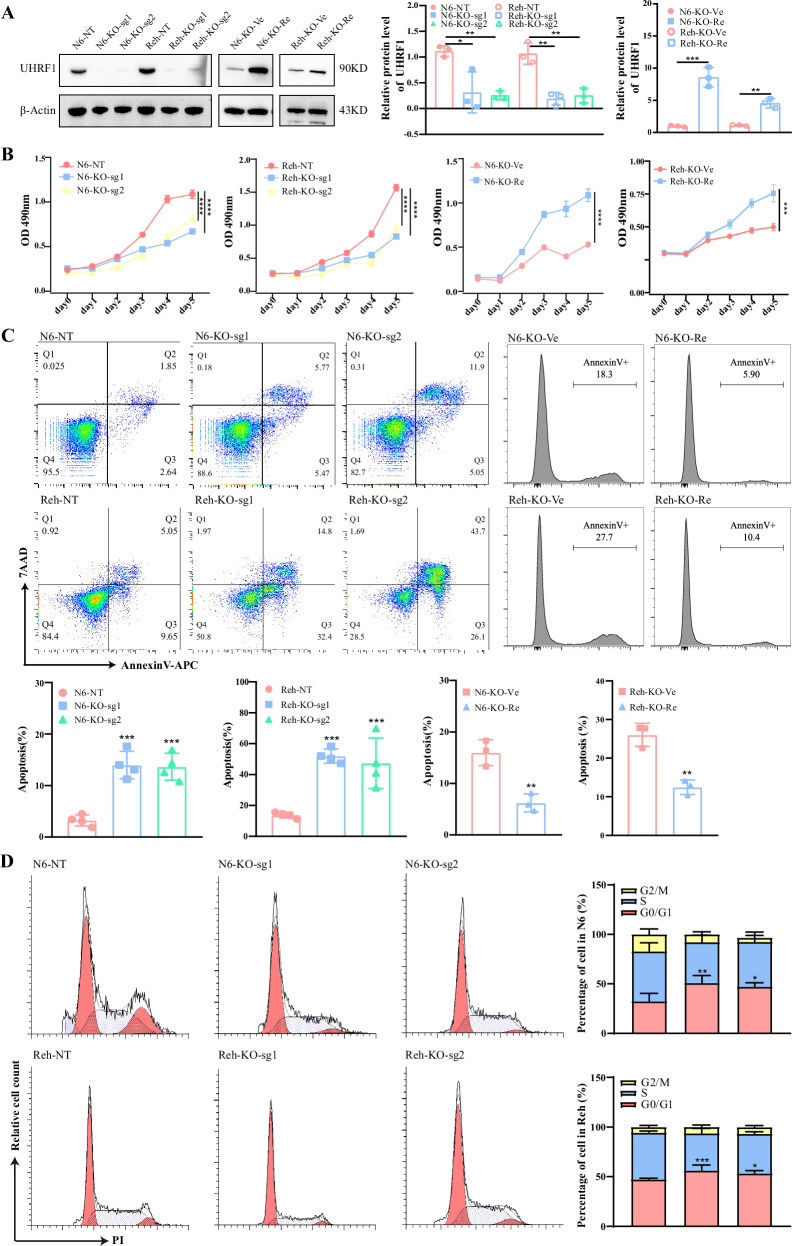
The impact of *UHRF1* on the biological phenotype of B-ALL cell lines. **A** The knockout and restoration efficiency of UHRF1 in Nalm6 and Reh cell lines was assessed via Western blot analysis. **B** The cell proliferation was detected using MTS methods. **C** and **D** The apoptosis and cell cycle of leukemia cells was detected using the flow cytometry. N6: Nalm6.

To investigate the function of *UHRF1* in B-ALL cell lines, CRISPR-Cas9 technology was utilized to knock out *UHRF1* in B-ALL cells, and a significant decrease in cell proliferation was observed (Figs. 2A, B and S3A). In the *UHRF1* rescue experiment, the proliferation rate of the rescue group was significantly higher than that of the control group (Figs. 2A, B and S3A). In the Nalm6 cell line, apoptosis increased by ~ 10% in the UHRF1 knockout group compared to the control group, while the percentage of cells in the G0/G1 phase increased by around 16%. Similarly, in the Reh cell line, apoptosis increased by about 35%, and the proportion of cells in the G0/G1 phase rose by ~8% in the UHRF1 knockout group compared to the control group. These findings suggested that UHRF1 knockout induces cell cycle arrest at the G0/G1 phase and enhances cellular apoptosis. Furthermore, restoration of UHRF1 expression reversed the increase in apoptosis (Fig. 2C, D).

### *UHRF1* regulates the expression of *SFRP5* through DNA methylation

**Fig. 3:**
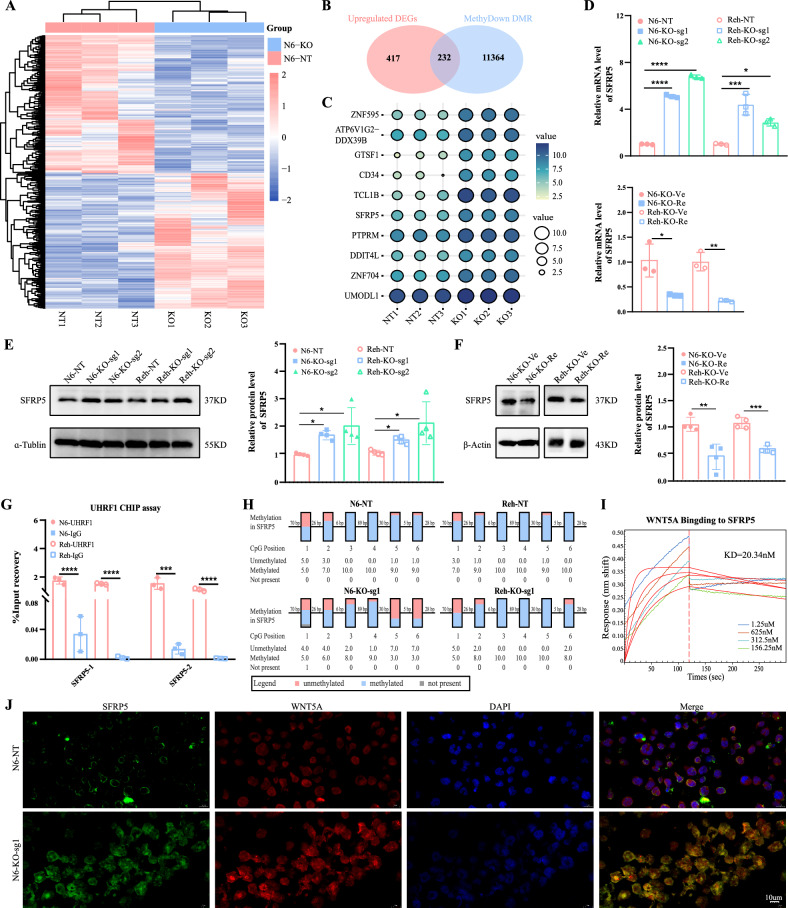
UHRF1 regulates the expression of SFRP5 through DNA methylation. **A** The heat map illustrates DEGs between the KO group and the NT group in the Nalm6 cell line. **B** In the KO group, the integration of up-regulated DEGs and demethylated DMR was analyzed by Wayne diagram. **C** Top 10 DEGs in the Intersection of Venn Diagram. **D** The mRNA expression level of *SFRP5* after *UHRF1* knockout and restoration. **E** and **F** The protein expression level of *SFRP5* after *UHRF1* knockout and restoration. **G** The CHIP-qPCR results indicate that the signal intensity of SFRP5 is stronger compared to the IgG control group. **H** The box plot demonstrates the upregulation of DNA methylation levels of SFRP5 following UHRF1 knockout. **I** Binding-dissociation curves between SFRP5 and WNT5A at different concentrations in BLI assay. **J**. Immunofluorescence staining reveals an increase in the protein levels of SFRP5 following UHRF1 knockout, and co-localization of SFPR5 and WNT5A on the cell membrane. The magnification of the images is ×850 for the full blots. N6: Nalm6.

To further elucidate the molecular mechanisms involved, RNA-seq analysis was performed in Nalm6 cells with and without *UHRF1* knockout. Using a false discovery rate (FDR) threshold of <0.05, and a log2|Fold Change| threshold of >0.5, 961 DEGs were identified, including 648 with upregulated expression and 313 with downregulated expression after *UHRF1* knockout (Fig. 3A). Given that *UHRF1* is a critical regulator of DNA, an 850k methylation array analysis was performed to further identify downstream target genes regulated by *UHRF1*. The results revealed significant hypomethylation in Nalm6 cells with *UHRF1* knockout. Using an FDR threshold of <0.05, and a |deltaBeta| threshold of >0.1, 11,019 differentially methylated regions (DMRs) were identified with reduced methylation levels in the *UHRF1* knockout group. A Venn diagram revealed 232 common DEGs (com DEGs) between the upregulated and hypomethylated DMRs in the *UHRF1* knockout group (Fig. 3B). The top ten genes were selected for subsequent validation (Fig. 3C). The RT-qPCR results showed that the mRNA expression level of *SFRP5* was significantly higher in the *UHRF1* knockout group compared to that in the control group but decreased after *UHRF1* restoration (Fig. 3D). The same result was confirmed at the protein level using western blot (Figs. 3E, F and S3B). The remaining 9 genes did not achieve consistent results. These results indicate that the expression level of *SFRP5* in B-ALL cells may be regulated by *UHRF1*-mediated DNA methylation modifications. To validate this hypothesis, the specificity of *UHRF1* protein binding to the promoter region of *SFRP5* was experimentally confirmed using ChIP-qPCR (Fig. 3H). Subsequently, bisulfite sequencing PCR was used to investigate the effect of *UHRF1* knockout on *SFRP5* DNA methylation. The results showed that among the six CpG sites in the promoter region of *SFRP5*, the methylation ratio was lower than that in the control group (Fig. 3G), suggesting that demethylation modifications occurred within *SFRP5* in the *UHRF1* knockout group. Furthermore, a statistically significant but weak negative correlation was observed between the mRNA expression levels of UHRF1 and SFRP5 in the TARGET dataset of pediatric B-ALL patients (Fig. S2C). Notably, this correlation is specific to pediatric samples and its biological implications should be interpreted with caution. The expression relationship between *SFRP5* and *UHRF1* should be examined in future studies using adult B-ALL datasets.

To further explore the clinical significance of *SFRP5*, peripheral blood, and bone marrow specimens were collected from 74 patient samples with B-ALL (27 with relapse, 40 newly diagnosed, and 7 in complete remission) and 25 healthy donors (HD) at our center for RT-qPCR analysis (Fig. S1C). As shown in Fig. S2D, *SFRP5* expression levels were significantly higher in HD compared to those in patients with relapsed and newly diagnosed. Furthermore, the expression levels of *SFRP5* in B-ALL patients who achieved CR tended to be higher than those in patients with relapsed or newly diagnosed cases.

Previous literature suggests that SFRP5 inhibits WNT5A, a β-catenin-independent WNT protein that plays a critical role in regulating cell signaling pathways involved in development and cancer progression. Notably, WNT5A has been implicated in various malignancies, including leukemia, and its dysregulation can contribute to tumorigenesis. Given the potential functional relationship between SFRP5 and WNT5A, we investigated their interaction to explore its relevance in the pathogenesis of B-ALL. In this study, immunofluorescence detection was performed in vitro, and the results suggested a co-localization of SFRP5 protein and WNT5A protein on the cell membrane (Fig. 3J). Furthermore, this simultaneously validated the increase in SFRP5 protein expression upon UHRF1 knockout. To validate the interaction between SFRP5 and WNT5A proteins, we employed BLI, a technique that allows real-time monitoring of biomolecular interactions without the need for labeling. BLI is particularly useful for assessing binding kinetics and affinity between proteins, making it ideal for studying protein-protein interactions. The results of the BLI assay showed a concentration-dependent binding of the WNT5A protein to the SFRP5 protein, with a measured KD value of 20.34 μM, indicating a strong binding affinity between the two proteins (Fig. 3I).

The above results indicate that *UHRF1* regulates the expression of *SFRP5* through methylation, and SFRP5 protein may affect the biological functions of B-ALL cells by inhibiting WNT5A protein.

### *UHRF1* regulates glycolysis via the P38 MAPK–HK2 signaling axis

**Fig. 4:**
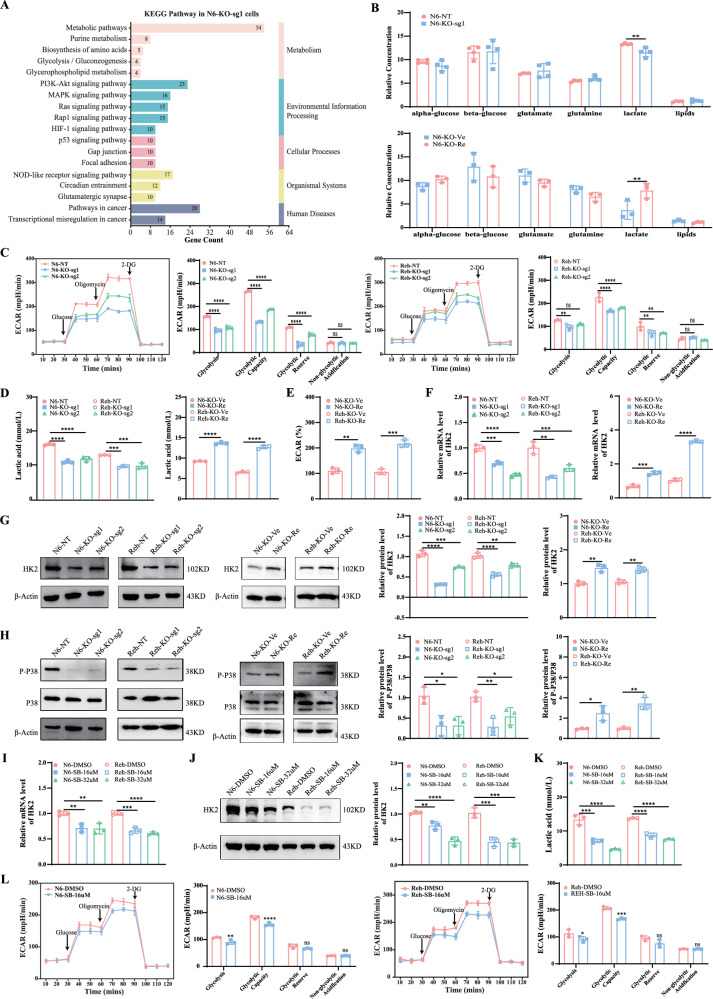
UHRF1 regulates glycolysis via the P38 MAPK-HK2 signaling axis. **A** The bar graph presents KEGG enrichment analysis results for DEGs in the transcriptome. **B** NRM-based metabolic analysis was conducted to investigate intracellular metabolites. **C** Representative ECAR profiles of Nalm6 and Reh cells following *UHRF1* knockout in the glycolytic rate assay. **D** Detection of lactate content in the cell culture medium. **E** Representative ECAR profiles of Nalm6 and Reh cells following *UHRF1* restoration in the extracellular acidification assay. **F** The mRNA expression level of *HK2* was detected by RT-qPCR. **G** Measurement of HK2 protein expression levels based on the western bot. **H** The phosphorylation level of P38 protein was assessed utilizing a western blot. **I** The mRNA expression level of *HK2* in B-ALL cell lines treated with P38 inhibitor (SB203580). **J** Comparing the expression levels of HK2 protein between groups treated with SB203580 and control groups. **K** The lactate content in the cell culture medium was decreased in B-ALL cell lines treated with SB203580. **L** Representative ECAR profiles of Nalm6 and Reh's cells following P38 inhibitor treatment in the glycolytic rate assay. N6 Nalm6, SB SB203580.

To further investigate how UHRF1 regulates the progression of B-ALL, we conducted a Kyoto Encyclopedia of Genes and Genomes (KEGG) enrichment analysis on the aforementioned 961 DEGs. The gene expression profiles related to metabolism, are primarily enriched in the purine metabolism, amino acid biosynthesis, and glycolytic pathway (Fig. 4A). Additionally, the PI3K-AKT and MAPK signaling pathways were involved. Therefore, metabolic reprogramming may occur during the progression of B-ALL.

To investigate changes in the metabolic products, cells from both the *UHRF1* knockout and rescue groups were collected for metabolomic analysis using nuclear magnetic resonance (NMR) spectroscopy. Substances were quantified based on the intensity of broad spectral bands encompassing categories such as amino acids, nucleic acids, lipids, glucose, and proteins. The results indicated that lactate levels in the UHRF1-knockout group were markedly lower than those in the control group, whereas the restoration of UHRF1 promoted an increase in lactate levels (Fig. 4B). These results were confirmed by lactate assay kits in vitro experiments (Fig. 4D). Furthermore, the bioenergetic profiles were examined using ECAR assay. The results revealed that knockout of *UHRF1* significantly decreased glycolysis and glycolytic capacity (Fig. 4C). Conversely, the restoration of *UHRF1* increased cell ECAR levels (Fig. 4E).

In analysis of the expression of glycolysis-related metabolic enzymes (such as *HK2*, *ENO1*, *GLUT1*) in B-ALL cell lines, RT-qPCR results only indicated a significant downregulation in *HK2* expression in the *UHRF1* knockout group (Fig. 4F). On the contrary, the expression level of *HK2* was markedly upregulated after *UHRF1* was restored (Fig. 4F). Correspondingly, the protein expression of *HK2* significantly decreased in the *UHRF1* knockout group and overexpressed in the *UHRF1* rescue group (Figs. 4G and S3C). To further clarify the signaling pathways regulating *HK2* expression, key proteins in the PI3K-AKT and MAPK signaling pathways were examined. The results showed that the absence of *UHRF1* had a pronounced inhibitory effect on the phosphorylation of the P38 protein (Figs. 4H and S3D). The rescue experiment verified the *UHRF1* role in the regulation of P38 protein phosphorylation (Figs. 4H and S3D). In addition, treatment of B-ALL cell lines with the P38 pathway inhibitor SB203580 resulted in noticeable downregulation of lactate level, glycolysis, glycolytic capacity, and *HK2* expression (Figs. 4I–L and S4A).

### SFRP5 downregulates glycolysis via the WNT5A-P38 MAPK-HK2 signaling axis

**Fig. 5:**
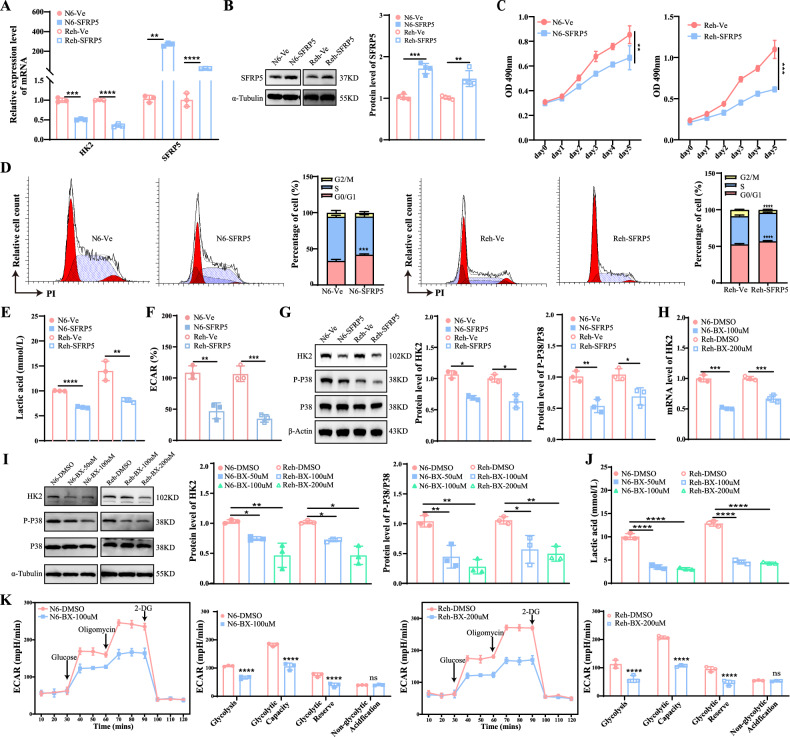
SFRP5 downregulates glycolysis via the WNT5A-P38 MAPK-HK2 signaling axis. **A** The mRNA expression level of *HK2* and *SFRP5* upon overexpression of *SFRP5* in B-ALL cell lines. **B** The protein expression level of SFRP5 upon overexpression of *SFRP5* in B-ALL cell lines. **C** The cell proliferation was detected using MTS methods. **D** The cell cycle of leukemia cells was detected using flow cytometry. **E** The lactate content in the cell culture medium was decreased in the *SFRP5* overexpression group. **F** Representative ECAR profiles of Nalm6 and Reh cells following *SFRP5* overexpression in the extracellular acidification assay. **G** Comparing the expression levels of phosphorylated P38 protein and HK2 protein between groups overexpressing SFRP5 and control groups. **H** The mRNA expression level of *HK2* in B-ALL cell lines treated with WNT5A inhibitor (Box 5). **I** Comparing the expression levels of phosphorylated P38 protein and HK2 protein between groups treated with BOX5 and control groups. **J** The lactate content in the cell culture medium was decreased in B-ALL cell lines treated with Box 5. **K** Representative ECAR profiles of Nalm6 and Reh cells following BOX5 treatment in the glycolytic rate assay. N6 Nalm6, BX BOX5.

The involvement of *SFRP5* in glucose and lipid metabolism, as well as the P38-MAPK signaling pathway, has been reported. However, whether *SFRP5* regulates the glycolytic pathway through the P38-MAPK pathway remains unclear. Overexpression of *SFRP5* in B-ALL cell lines resulted in a significant downregulation of *HK2* expression and the phosphorylation of the P38 protein (Figs. 5A, B, G and S4B, S4C). Overexpression of *SFRP5* inhibited cell proliferation in both Nalm6 and Reh cell lines (Fig. 5C). In the Nalm6 cell line, the percentage of cells in the G0/G1 phase increased by ~8% in the *SFRP5* overexpression group. Similarly, in the Reh cell line, the proportion of cells in the G0/G1 phase increased by about 7% in the *SFRP5* overexpression group compared to the control group (Fig. 5D). Additionally, both lactate and ECAR levels were reduced following *SFRP5* overexpression (Fig. 5E and F).

The inhibitor of WNT5A, BOX5, was used for in vitro intervention in B-ALL cell lines. The results showed that the expression of HK2 was significantly suppressed, and the phosphorylation of the P38 protein was reduced. (Figs. 5I, J and S4D) Furthermore, the lactate level, glycolysis, and glycolytic capacity were significantly decreased following BOX5 was applied in both Nalm6 and Reh cells, confirming the impact of BOX5 on glycolysis metabolism (Fig. 5K).

**Fig. 6:**
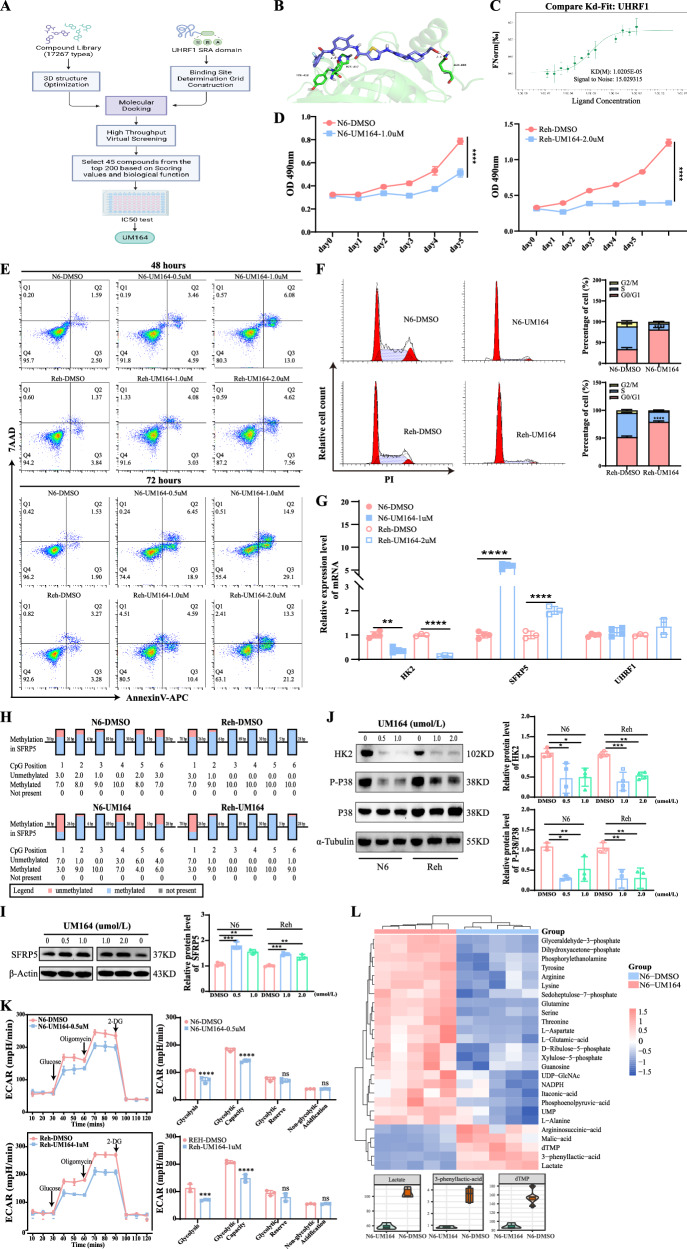
*UHRF1* inhibitor UM164 displays anti-leukemia activity in vitro. **A** The flowchart of the compound virtual screening process. **B** Molecular interaction diagram between UHRF1 protein and UM164 compound. **C** MST analysis of binding affinity between UHRF1 protein and UM164 compound. **D** The cell proliferation of B-ALL cell lines treated with UM164. **E** and **F** The apoptosis and cell cycle of leukemia cells treated with UM164 were detected using flow cytometry. **G** The mRNA expression level of *HK2*, *SFRP5,* and *UHRF1* in B-ALL cell lines treated with UM164. **H** The box diagram of DNA methylation status. **I** The protein expression levels of SFRP5. **J** The expression levels of phosphorylated P38 protein and HK2 protein. **K** Representative ECAR profiles of Nalm6 and Reh cells following UM164 treatment in the glycolytic rate assay. **L** The hierarchical heatmap of differential metabolites(up) and the violin plot of differential metabolites(down). N6 Nalm6.

Therefore, the above studies elucidated the involvement of *UHRF1* in the progression of B-ALL via the SFRP5/WNT5A -P38 MAPK-HK2 axis (Fig. 6).

### UM164 inhibits the survival of B-ALL cells in vitro by targeting *UHRF1*

To identify potential candidate inhibitors targeting UHRF1, structure-based virtual screening was performed (Fig. 6A). Molecular docking analysis using the Maestro module within the Schrödinger software suite was conducted on the crystal structure of UHRF1 (PDB accession code: 6VCS) (Fig. 6B). A total of 45 candidate compounds were subjected to in vitro validation, and the antileukemic activity of UM164 was confirmed using an IC50 assay. The docking confirmation revealed that UM164 established both hydrogen and π-bond interactions within the SRA domain groove of UHRF1, engaging with HIE417 and GLN606 residues. The microscale thermophoresis (MST) analysis showed that UM164 illustrated good affinity to UHRF1 (KD = 10.2 µM, Fig. 6C). In vitro experiments demonstrated that UM164 inhibited proliferation and induced apoptosis of B-ALL cells in a concentration- and time-dependent manner (Fig. 6D, E). Additionally, UM164 significantly induced cell cycle resetting in B-ALL cells (Fig. 6F). Bisulfite sequencing PCR (BSP) analysis revealed that UM164 treatment induced demethylation of the promoter region of *SFRP5* (Fig. 6H). UM164 upregulated SFRP5 expression without altering UHRF1 expression (Figs. 6G, I, and S4E). However, it reduced HK2 expression and suppressed the phosphorylation of the P38 protein (Figs. 6G, J, and S4F). Additionally, the ECAR assay demonstrated significantly reduced glycolysis and glycolytic capacity in leukemia cells treated with UM164 compared to the control group (Fig. 6K). Mass spectrometry analysis of energy metabolism further revealed a marked decrease in the levels of lactate, 3-phenyllactic acid, and dTMP in the UM164-treated group relative to the control group (Fig. 6L).

### UM164 delays the progression of B-ALL cells in vivo

**Fig. 7:**
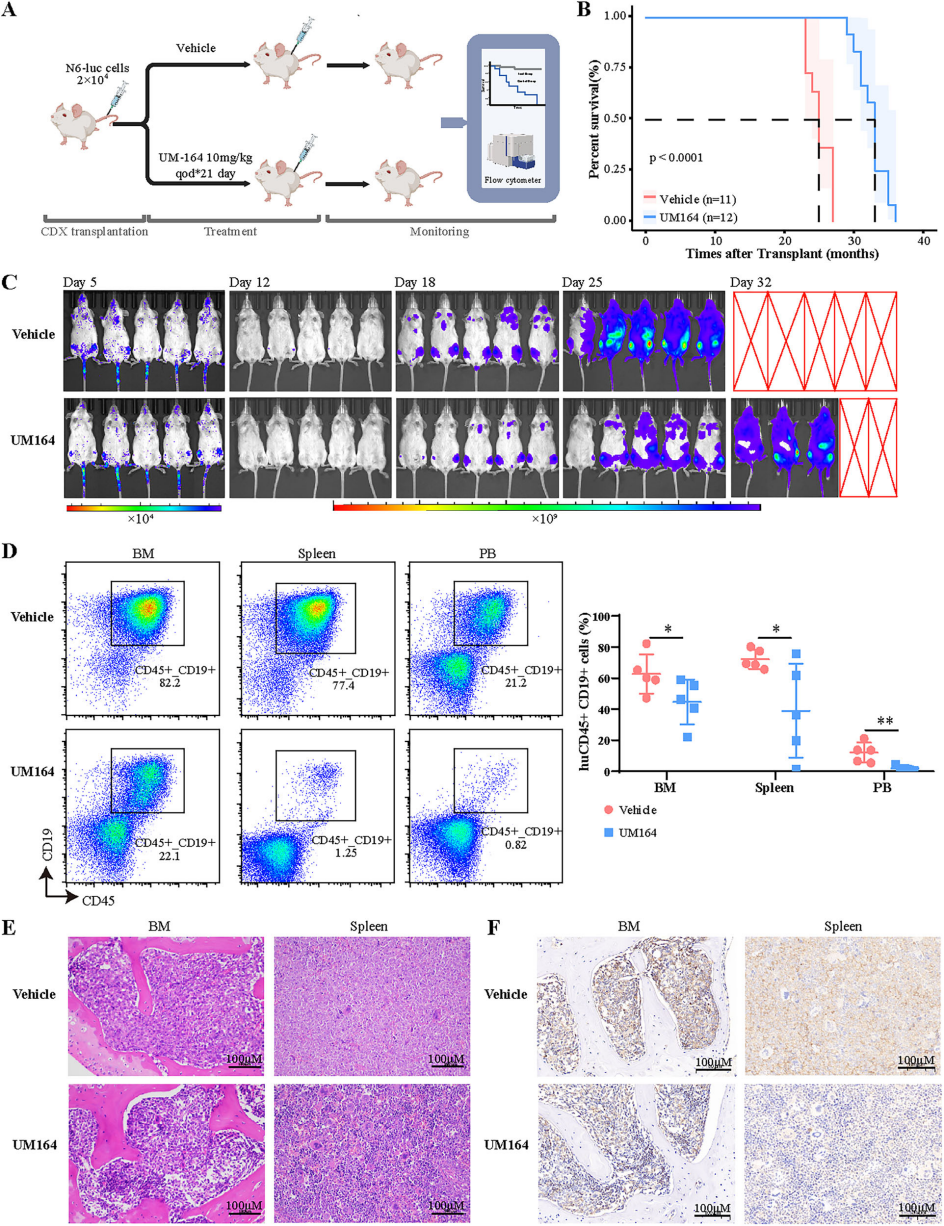
UM164 inhibits leukemia cell progression in vivo. **A** The process diagram for Nalm6-luc cells xenograft model construction and UM164 intervention therapy. **B** The survival of mice treated with the vehicle or UM164. **C** Bioluminescence imaging technology to evaluate the anti-leukemia effect of UM164 treatment. **D** The flow cytometry analysis of human CD45^+^CD19^+^ ratio in bone marrow, spleen, and peripheral blood cells isolated from the vehicle- or UM164-treated mice transplanted with Nalm6-luc cells (*n* = 5). **E** and **F** The HE stains analysis and immunohistochemical analysis of CD45 in spleen and BM cells isolated from the vehicle- or UM164-treated mice were performed 3 weeks after transplantation. The magnification of the images is ×200 for the full blots. N6-luc Nalm6-Luciferase.

To investigate the therapeutic effects of UM164 on B-ALL in vivo, we transplanted Nalm6 cells into Non-obese diabetic (NOD)/ShiLtJGpt-*Prkdc*^*em26Cd52*^*Il2rg*^*em26Cd22*^/Gpt (NCG) mice (Fig. 7A). UM164 treatment, initiated 5 days after transplantation, delayed leukemia progression and significantly extended survival time, increasing median overall survival (OS) from 25 to 33 days without inducing body weight loss (Fig. 7B). Leukemia progression was monitored using bioluminescence imaging (Fig. 7C). Three weeks post-transplantation, analyses of bone marrow, spleen, and peripheral blood revealed a reduced presence of CD45^+^ CD19^+^ leukemia cells in UM164-treated recipients (Fig. 7D). Morphological and immunohistochemical analyses further demonstrated a substantial reduction in leukemia cell infiltration within the spleen and bone marrow of UM164-treated mice compared to controls (Fig. 7E and F). Together, these findings highlight the therapeutic potential of UM164 in treating B-ALL, both in vitro and in vivo.

## Discussion

Relapsed/refractory B-ALL is characterized by increased aggressiveness and poor prognosis. In adult B-ALL patients, the relapse rate is high, with more than 50% of patients experiencing relapse after achieving complete remission. Despite the emergence of several targeted therapeutic approaches showing favorable outcomes in select cases, a significant proportion of individuals still experience unfavorable responses. The prognosis for these patients remains poor, with a 5-year overall survival (OS) rate ranging from only 3–10% [17]. The mechanisms governing the evasion of RR B-ALL from established treatment strategies remain unclear.

*UHRF1* is a key epigenetic regulator involved in cell division [18]. It critically contributes to the transition from the G1 to S phase in the cell cycle in several cancers, including breast cancer, bladder cancer, lung cancer, and colorectal cancer [19]. Research has clarified *UHRF1*’s pivotal role in mediating the silencing of several tumor suppressor genes, including *p16INK4A*, *hMLH1*, *p21*, and *RB*, laying the foundation for subsequent carcinogenesis [20]. *UHRF1* overexpression is associated with increased aggressiveness of tumors in renal cell carcinoma and acute myeloid leukemia cells [21, 22]. Our investigations unveiled a marked elevation in *UHRF1* expression in RR B-ALL, concomitant with an inadequate prognosis. Therefore, we hypothesized that *UHRF1* is involved in the pathogenesis of B-ALL. The experimental findings revealed a decrease in the proliferation of B-ALL cells after deletion of *UHRF1*. Furthermore, the knockout of *UHRF1* correlated with cell cycle arrest and increased apoptosis. These findings are consistent with Hanash’s research that *UHRF1* regulated cell cycle and apoptosis in B-ALL cells [23].

The aberrant expression of *UHRF1* is implicated in the remodeling of the epigenetic landscape [24]. DNA hypermethylation extensively influences the leukemogenesis and progression of ALL, and it is correlated with adverse prognosis [13, 25, 26]. Our investigation has verified that knockout of *UHRF1* leads to a widespread decrease in DNA methylation levels. The methylation level of the *SFRP5* DNA promoter region is directly regulated by *UHRF1*. Previous studies have reported that DNA hypermethylation of secreted frizzled-related proteins (*SFRPs*) is commonly observed in leukemia patients, resulting in the downregulation of *SFRP* expression. This phenomenon is particularly pronounced in Philadelphia chromosome-positive B-ALL and serves as an independent adverse prognostic factor [27, 28]. In a study on acute myeloid leukemia, elevated methylation of *SFRP5* was observed to upregulate MDR1 expression, leading to the development of multidrug resistance [29]. Moreover, the abnormal expression of *UHRF1* and *SFRP5* is associated with tumor recurrence [30, 31].

In this study, overexpression of *SFRP5* suppressed the proliferation of B-ALL cell lines. Thus, the question of how *UHRF1* and *SFRP5* participate in the process of relapse and refractory challenges in B-ALL was raised. Weng Jieming et al. found that *UHRF1* modulated glucose glycolysis and lipid metabolism by regulating AMPK activity [32]. Additionally, *SFRP5* is primarily produced and secreted by adipose tissue, exhibits anti-inflammatory characteristics, and plays multifaceted roles in metabolism [33–36]. *SFRP5* also participates in lipid metabolism and energy balance and serves as an important mediator in the regulation of glucose homeostasis and insulin sensitivity. Moreover, *SFRP5* inhibits mitochondrial oxidative metabolism, thereby promoting adipocyte differentiation [37]. *SFRP1* is involved in the regulation of aerobic glycolysis via the Wnt signaling pathway [38, 39]. In addition, *SFRP2* and *SFRP4* substantially enhanced glycolysis in cardiac fibroblasts and myotubes separately [40, 41]. However, there are currently no published studies on the relationship between *SFRP5* and glycolysis.

Here, single-cell sequencing of clinical samples and RNA-seq analysis of cell lines revealed a close association between *UHRF1* and metabolic pathways, including glycolysis. In vitro, experimental results demonstrated that silencing *UHRF1* and overexpression of *SFRP5* both reduced lactate levels, inhibited the phosphorylation of the P38 protein, and significantly decreased the expression of the key glycolysis enzyme *HK2*. The WNT5A protein, targeted by SFRP5 for inhibition, can activate the phosphorylation level of P38 and participate in the regulation of the glycolytic pathway independently of β-catenin [42–45]. In this study, immunofluorescence demonstrated co-localization of WNT5A and SFRP5 on the cell membrane, further confirmed by BLI to exhibit a strong binding affinity between the two proteins. Furthermore, the WNT5A inhibitor was demonstrated to reduce lactate production and downregulate the phosphorylation of the P38 and HK2 proteins. Additionally, both our study and the existing literature show that inhibition of P38 can downregulate the expression of *HK2*, thereby attenuating aerobic glycolysis [46, 47].

We employed virtual compound library screening to identify the inhibitor UM164 that targets *UHRF1*, which has been validated through in vitro experiments to induce apoptosis, suppress proliferation, and induce cell cycle arrest in B-ALL cells. In vivo experiments indicated that UM164 attenuated the progression of B-ALL in murine CDX models. Interestingly, UM164 hindered the phosphorylation of P38 protein, diminished the expression of *HK2*, and concurrently led to a decrease in lactate content within cells post-UM164 treatment in vitro studies using cell lines. Additionally, UM164 disrupted the preservation of DNA methylation at the *SFRP5* promoter, consequently triggering the activation of *SFRP5* in B-ALL cells. These results confirm that UM164 affects the expression of *SFRP5* through methylation modifications, thereby participating in the regulation of glycolysis through the P38 MAPK-HK2 signaling axis. Hence, targeting the DNA methylation function of *UHRF1* via UM164 is a potential therapeutic strategy, which could improve the prognostic outlook of RR B-ALL.

It is important to acknowledge the limitations of this study. The small sample size of the sequencing data may impact the generalizability and robustness of the findings. Besides, the TARGET dataset predominantly contains pediatric B-ALL cases. While pediatric and adult B-ALL share certain genetic and molecular features, there are notable differences in disease biology, prognosis, and treatment responses between these populations. These distinctions should be considered when extrapolating findings from the TARGET dataset to adult B-ALL. Future efforts to establish and share adult B-ALL datasets would be invaluable for advancing research in this field. Lastly, only the Nalm6 cell line represents adult B-ALL, whereas the Reh cell line is derived from pediatric B-ALL.

In summary, our investigation suggests that the epigenetic regulator *UHRF1* is involved in the progression of B-ALL by modulating the WNT5A–P38 MAPK–HK2 signaling axis, which mediates the glycolytic pathway. Targeted intervention of *UHRF1* holds promise for halting the progression of B-ALL.

## Supplementary information

Supplementary Figures

## Data Availability

Sequencing data have been deposited at the National Omics Data Encyclopedia (NODE, https://www.biosino.org/node/). Further information and requests for raw data should be directed to and will be made available by the lead contact Jianda Hu (drjiandahu@163.com) upon reasonable request.
