# Correction: *UHRF1*-mediated epigenetic reprogramming regulates glycolysis to promote progression of B-cell acute lymphoblastic leukemia

**DOI:** 10.1038/s41419-025-08136-4

**Published:** 2026-01-13

**Authors:** Yan Huang, Luting Luo, Yangqi Xu, Jiazheng Li, Zhengjun Wu, Chenxing Zhao, Jingjing Wen, Peifang Jiang, Haojie Zhu, Lingyan Wang, Yanxin Chen, Ting Yang, Jianda Hu

**Affiliations:** 1 Fujian Medical University Union Hospital, Fuzhou, Fujian PR China; 2 The Second Affiliated Hospital of Fujian Medical University, Quanzhou, Fujian PR China; 3 Institute of Precision Medicine, Fujian Medical University, Fuzhou, Fujian PR China; 4 Zhangzhou Affiliated Hospital of Fujian Medical University, Zhangzhou, Fujian PR China; 5 Department of Immunology, School of Basic Medical Sciences, Fujian Medical University, Fuzhou, Fujian PR China; 6 Department of Lymphoma, Fujian Medical University Cancer Hospital, Fujian Cancer Hospital, Fuzhou, Fujian PR China; 7 The Second Department of Hematology, National Regional Medical Center, Binhai Campus of the First Affiliated Hospital, Fujian Medical University, Fuzhou, Fujian PR China

**Keywords:** Acute lymphocytic leukaemia, Mechanisms of disease

Correction to: *Cell Death and Disease* 10.1038/s41419-025-07532-0, published online 29 April 2025

During the submission of the final version of the manuscript, an error occurred during figure layout resulting in the lower right H&E-stained image in Fig. 7E being a duplicate of the upper right image.

**Original Figure 7** 
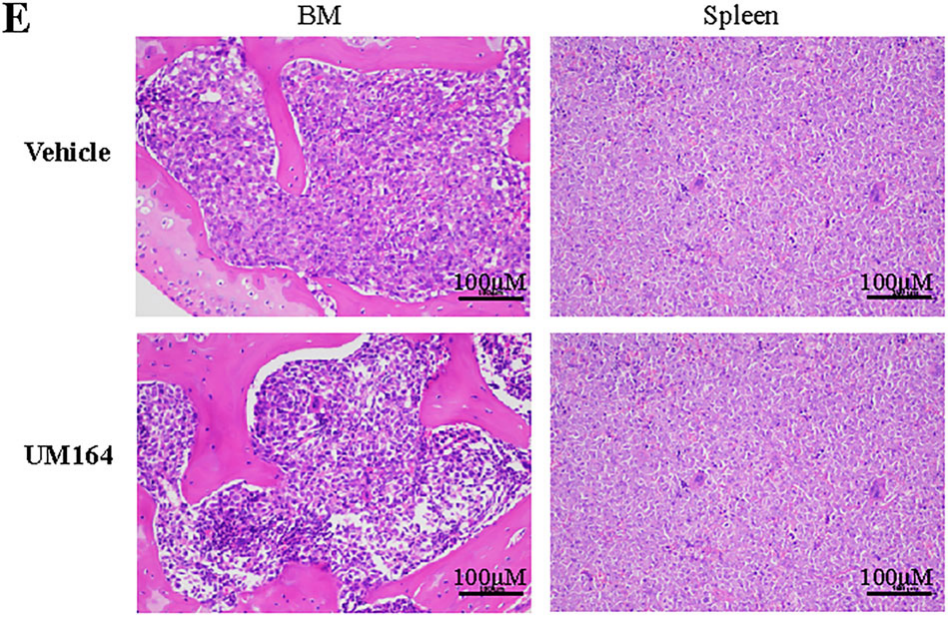


**Amended Figure 7** 
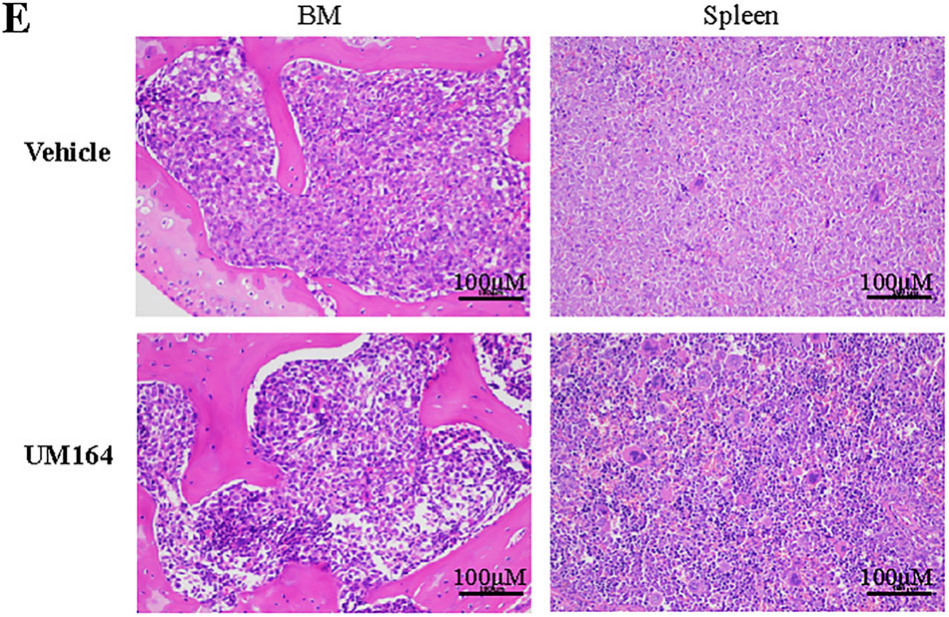


The original article has been corrected.

