# Supplementary material for: UHRF1-mediated epigenetic reprogramming regulates glycolysis to promote progression of B-cell acute lymphoblastic leukemia
Source: Cell Death Dis. 2025 Apr 29;16(1):351. doi: 10.1038/s41419-025-07532-0 (PMC12041315; doi:10.1038/s41419-025-07532-0)
Supplement: Supplementary file 1 — Supplementary Figures [file 41419_2025_7532_MOESM1_ESM.docx]

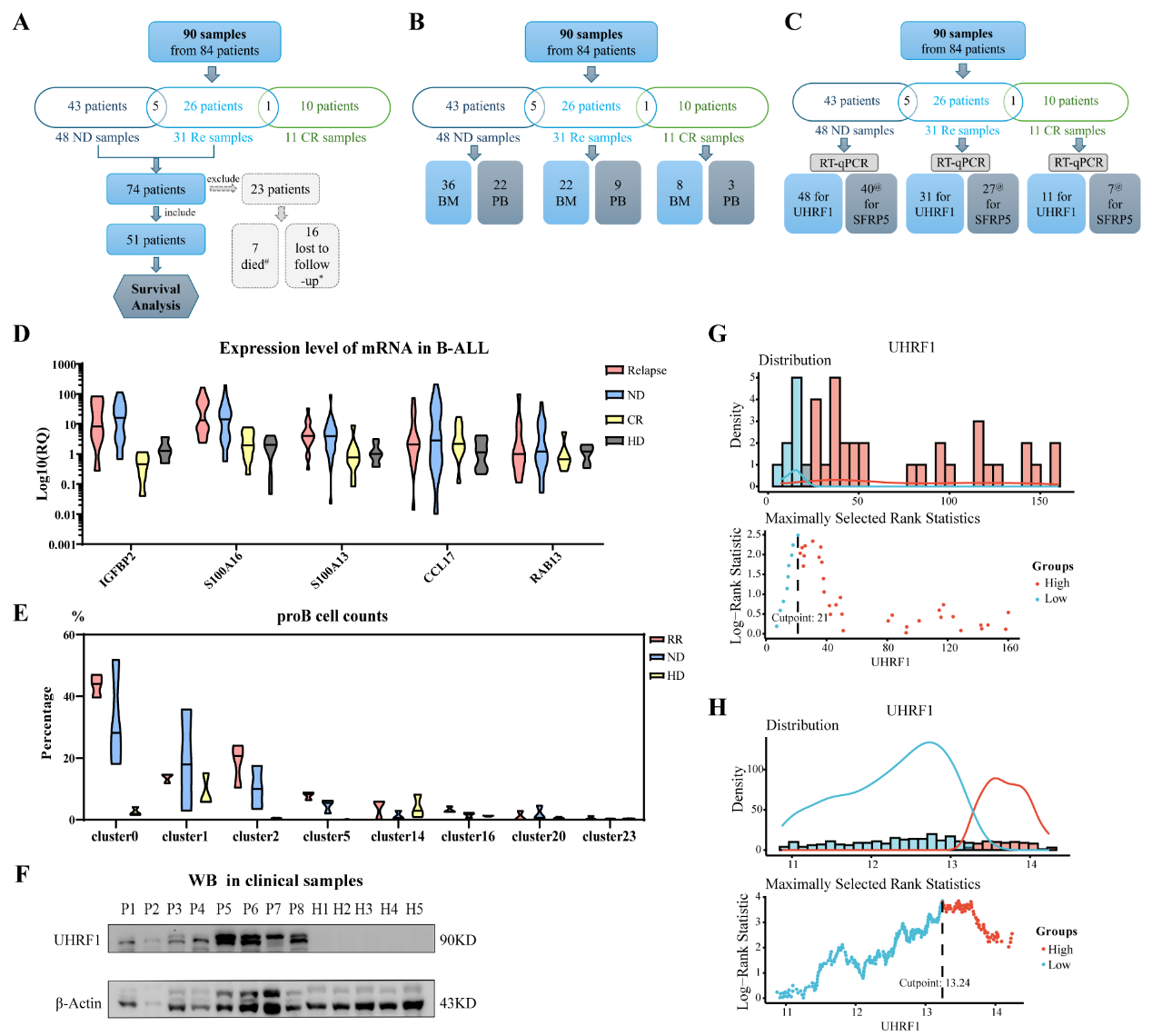


**Figure S1. The expression levels of *UHRF1* and other common DEGs in B-ALL patients.**

A-C. The flow chart of the selection in patients. D. The proportion of progenitor B-cell in three groups using scRNA-seq. E. The mRNA expression levels of the remaining five common DEGs in B-ALL Patients across varied disease states. F. Comparative evaluation of UHRF1 protein expression levels between B-ALL patients and healthy individuals. G. The optimal cutoff value of *UHRF1* expression with B-ALL patients from our institution's dataset was determined using the log-rank function. H. The optimal cutoff value of *UHRF1* expression with B-ALL patients from the TARTGET dataset was determined using the log-rank function.

Re: Relapse, CR: Complete Remission, RR: Relapsed/Refractory, ND: Newly Diagnosed, HD: Healthy Donor, BM: Bone Marrow, PB: Peripheral Blood.

^#^7 patients died during the first induction remission treatment.

^*^16 patients were lost to follow-up after receiving one or two chemotherapy treatments.

^@^ Sixteen of 90 samples were excluded due to a lack of available specimens

**
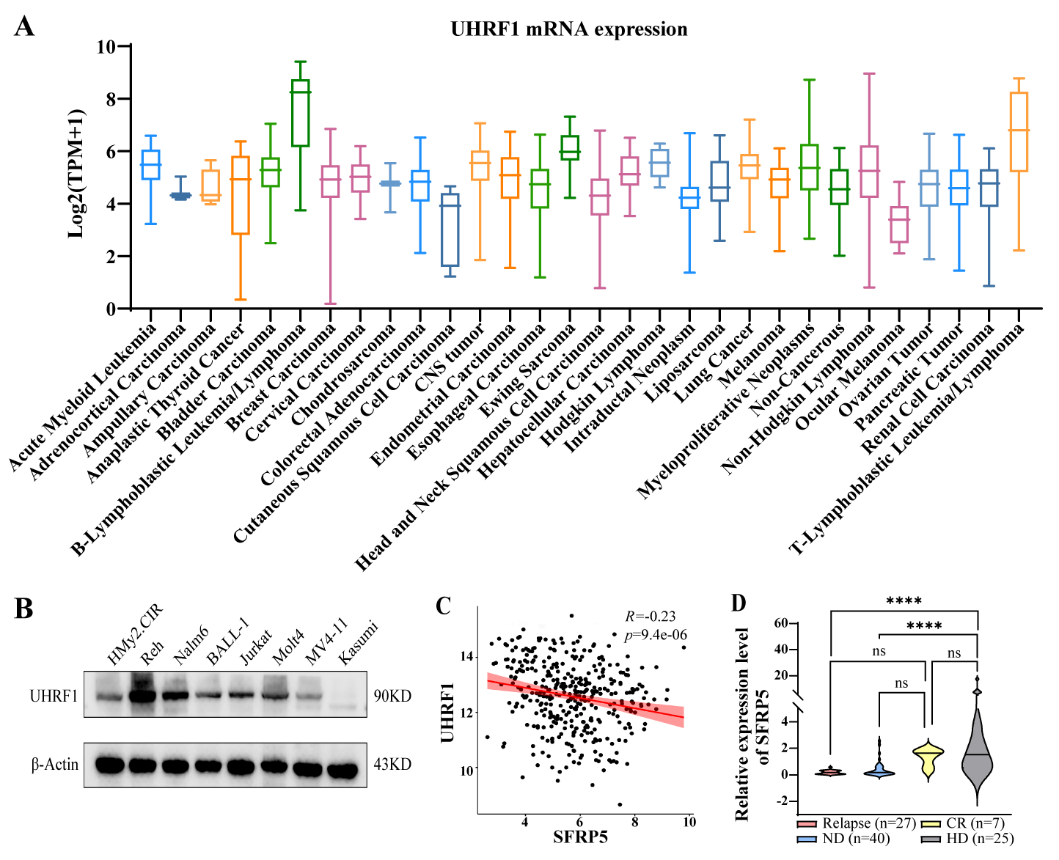
**

**Figure S2. The expression level of *UHRF1* in tumor cell lines and the expression levels of *SFRP5* in B-ALL patients.**

A. The mRNA expression of *UHRF1* in different tumor cell lines. B. Protein expression of UHRF1 in different cell lines. C. The mRNA expression levels of *UHRF1* and *SFRP5* in the B-ALL dataset from TARGET database were negatively correlated. D. The mRNA expression levels of *SFRP5* were significantly lower in B-ALL patients compared to healthy donors (HD). Among B-ALL patients, *SFRP5* expression levels tended to be higher in those who achieved complete remission compared to patients with relapse or newly diagnosed cases.

ND: Newly Diagnosed group, CR: Complete Remission group, HD: Healthy Donor group.


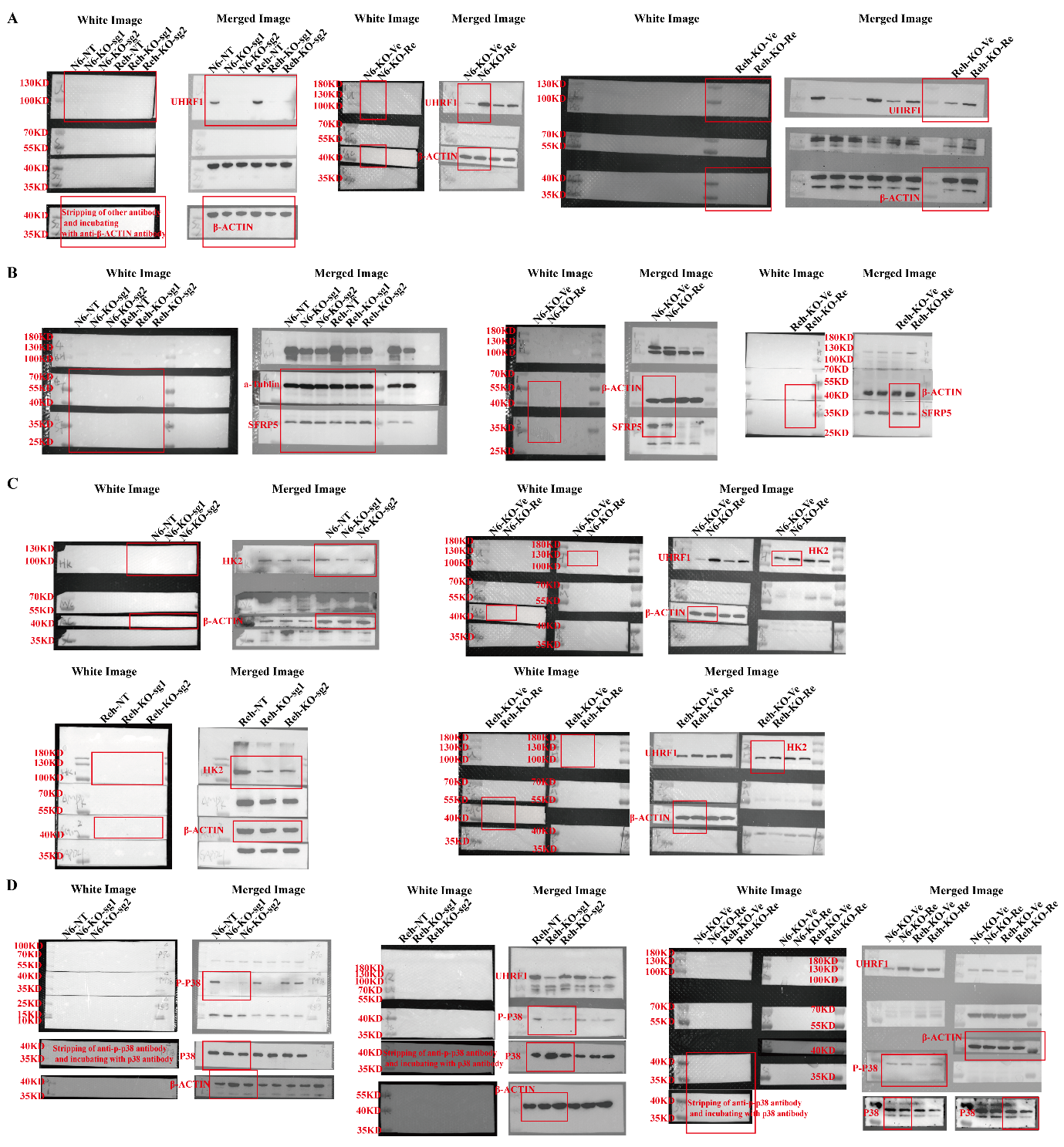


**Figure S3. The original WB images after the knockout and restoration of UHRF1.**


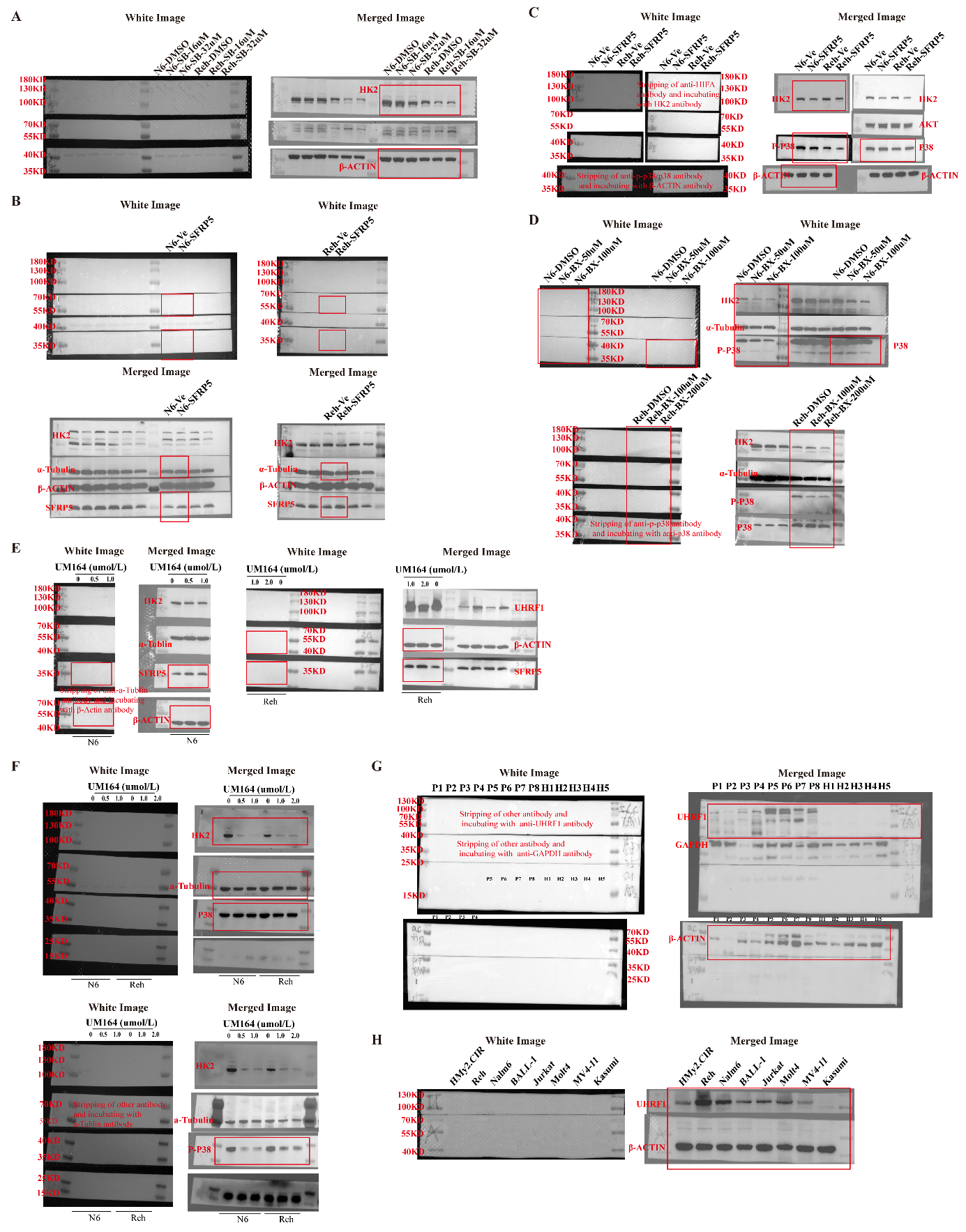


**Figure S4. The original WB images after the overexpression of SFRP5, and B-ALL cell lines treated by SB202038, BOX5 and UM164.**
